# The habit, choice, intention, and perception of raw beef consumers on raw beef-eating: the health risk management perspective

**DOI:** 10.1186/s40795-022-00564-1

**Published:** 2022-07-25

**Authors:** Daniel Teshome Gebeyehu, Biruk Alemu, Gemechu Belete

**Affiliations:** 1 School of veterinary medicine, Wollo University, Dessie, Ethiopia; 2 Department of psychology, teachers and behavioral science institute, Wollo University, Dessie, Ethiopia

**Keywords:** Raw beef eating, Habit, Choice, Intention, Perception, Raw beef consumers

## Abstract

Apart from its nutritive value, meat is one of the substances for the transmission of pathogenic micro-organisms to consumers and the raw beef eating habit of Ethiopians can create a favourable condition for the transmission of pathogens from contaminated meat to raw beef consumers. The face-to-face interview of raw beef consumers was done using a structured questionnaire and 570 total samples were collected. A considerable number (74%) of raw beef consumers had favourable food choice; 85% of the raw beef consumers had favourable intentions to stop their raw beef eating habit, and 67% of them had an unfavourable perception of the safety of raw beef-eating. In conclusion, the study showed that raw beef consumers were not aware of the health risks of raw beef-eating. As a result, urgent sensitization intervention is required to shift the raw beef consumers from unhealthy eating habits to prudent (processed) eating practices.

## Introduction

Meat is a potential source of biological protein and essential nutrients [1]. Apart from its nutritional and health benefits, meat can be a source of both chronic [2] and infectious diseases [3]. The infectious diseases that originate from meat can be prevented using proper heat and cold treatments [4].

Even though modern technologies are advanced for safe meat production, the safety of meat processing in developing countries including Ethiopia is still a concern. Except in the big cities of Ethiopia, the animals are slaughtered locally in open areas without any hygienic prerequisites [5]. As a result, the chance of the meat being contaminated with pathogenic microbes is exceedingly high. The contamination of meat occurs during the removal of hides, evisceration, processing, packaging and storage, and distribution at slaughterhouses and retail outlets [6]. Microorganisms that contaminate meat not only predispose to spoilage but also spread food-borne illness to consumers [6].

Not only processed (cooked, roasted, stewed, and fried) meat, eating raw beef is commonly practiced throughout Ethiopia. Besides beef, eating raw meat from other animals is not common [7]. The raw beef in Ethiopia is called “Kurt” in the Amharic language. “Kurt” is directly consumed without any process by mixing with hot pepper and other locally prepared spices. Except the studies conducted on the meat-eating culture [7] and the raw beef eating preference of consumers [8], no study was conducted on the current or related topics and there is no written document available about how this raw beef-eating practice was began, but there is a verbal story that describes eating raw beef began during wartime when soldiers did not have access to fire and had limited time for cooking. The raw beef-eating habit of Ethiopians can create a favourable condition for pathogens to pass from contaminated meat to raw beef consumers [9] and this eating habit is suggested to be changed for the sake of reducing health crises from foodborne pathogens.

To the best of our knowledge, the study on the raw beef-eating habit, choice, perception, and practice of raw beef consumers or its similar was not done in Ethiopia or elsewhere. As a result, this study will be the first for investigating the raw beef consumers’ eating behavior. Not only eating behavioral change, it is important to formulate implementable and consumer-oriented meat safety regulation [10]. Since there is no previous study on our topic of interest, we were depending on a research hypothesis: raw beef consumers’ food choice is raw beef, raw bee consumers are not intended to stop raw beef eating, and they have positive perception on the safety of raw beef eating. To change the raw beef eating behavior and to formulate appropriate food safety regulation, it is imperative to assess consumers’ habits, choice, intention, and perception. Therefore, this study was conducted with the objective of assessing the eating habits of raw beef consumers, the consumers’ choice of raw beef-eating, consumers’ intention toward reducing/stopping raw beef consumption, and their perception of the safety of raw beef-eating.

## Materials and methods

### Study area

The study was conducted in selected places in South Wollo (Dessie, Kombolcha, and Wereilu), and Oromia (Kemissie and Bati) zones. South Wollo and Oromia zones are in the Amhara regional state with the geographic coordinates of 10.8997° N, 38.9877° E, and 10.3959° N, 40.0000° E, respectively. South Wollo and Oromia zones are situated in the north-eastern part of Ethiopia, 401, and 327 km away from Addis Ababa (the capital city of Ethiopia), respectively. South Wollo and Oromia zones cover the area of 17,067.45 km^2^, and 286,612 km^2^, respectively.

### Study population

South Wollo and Oromia zones have a total population number of 2,518,862 and 457,278 respectively [11]. The study population was consumers of raw beef in selected raw beef restaurants. For the eating behavior assessment, all age groups greater than 18 years old and both sexes were included. A total of 570 raw beef consumers were interviewed. The majority (70.18%) of the participants were from South Wollo (35.09% in Dessie, 26.32% in Kombolcha, and 8.77% in Wereilu) and the remaining (29.82%) were from the Oromia zone (17.54% in Kemissie, and 12.28 in Bati).

### Study design

A cross-sectional type of study (a study that investigates a situation at a point in time) was carried out from January 2021 to September 2021 in selected cities and towns of South Wollo and Oromia zones for assessing the raw beef-eating behavior of raw beef consumers. In this study, both descriptive and inferential statistics were used.

### Sample size and data collection techniques

The sample size for eating behavior was done based on the suggestions of Taherdoost’s formula [12]. Taherdoost and his research team suggested that for every type of cross-sectional survey the following formula is more appropriate than others.

$$n=\frac{p\ \left(100-p\right)\ {z}^2}{e^2}$$

Where n = is the required sample size.

p = is the percentage occurrence of a state or condition.

z = is the value corresponding to the level of confidence required.

e = is the percentage maximum error required.

Since there was no preceded raw beef-eating behavior assessment conducted in the study areas, 50% for *p*-value, 95% (1.96) for z-value, and 5% for e-value were taken. As a result, the sample size was calculated as follows.

$$n=\frac{50\left(100-50\right){1.96}^2}{5^2}$$

$$=384\;\mathrm{minimum}\;\mathrm{samples}\;\mathrm{were}\;\mathrm{required}$$

Even if the minimum sample size is 384, the researchers collected a higher number of samples (570). The total sample size from Dessie, Kombolcha, Kemise, Bati, and Werielu were 200, 150, 100, 70, and 50, respectively.

Structured questionnaire interviews were conducted to assess the raw beef-eating behavior of raw beef consumers. The tables in the randomly selected raw beef restaurants were chosen randomly and any raw beef consumer in the selected table of each restaurant was invited for an interview. All the selected restaurants have sold both raw and processed (roasted, cooked, and fried) beef. Only raw beefeaters in the raw beef restaurants at the time of the interview who were volunteering to be interviewed were used and processed meat consumers were excluded. Those raw beef consumers who were not volunteer for an interview in the selected table were excluded from sampling. Lunchtime was purposively selected for the interview and one raw beef consumer was interviewed from 30 minutes to 1 hour depending on how fast the raw beef consumer understood the questions. The interview continued until the data or information saturation was attained. All the questions in the questionnaire were close-ended. The questionnaire has five sections and different sets of questions. The first section was about the general demographic characteristics of the raw beef consumers and the second section of the questionnaire was about the general raw beef eating habit of raw beef consumers while the third and fourth sections were about the choice of raw beef for consumption and their intention to change or minimize raw beef-eating, respectively. The fifth section of the questionnaire was about the perception of raw beef consumers toward raw beef-eating. The questions in sections three, four, and five enabled the researcher to understand the choice, intention, and perception of raw beef consumers, respectively. The questionnaire was composed of 34 questions/variables. Seven questions were used for each section of demographic characteristics, and general eating habits, eight questions about the perception of eating raw beef  and six questions were used for each choice and intention of raw beef consumers.

All the questions concerned on the choice, intention, and perception of the raw beef consumers were pooled into a single variable, which had two categories. These two categories were favourable or unfavourable for choice, and favourable or unfavourable for both intention and perception of the raw beef consumers.

The consumers’ choice of raw beef eating was assessed based on the food choice conceptual model [13]. Six questions that are related to the consumers food preparation preference, the food type usually consumed, reason for the usual consumption of specific food item, feelings if consumers did not eat the usual food item, daily frequency of eating the usual food item, and the mealtime consumers eat their usual food item.

The intention of the raw beef consumers was assessed based on the theory of planned behavior [14]. Six questions (intention to reduce raw beef eating, knowledge on the health risk of raw beef-eating, intention to improve their knowledge on raw beef-eating health risk, willingness to stop raw beef-eating if consumers know raw beef-eating health risk, easiness to stop raw beef-eating, and obstacles to stop raw beef-eating) were used to investigate the raw beef consumers’ intention to stop eating raw beef.

The perception of raw beef consumers towards the safety of raw beef-eating was assessed based on Likert’s scale [15]. The agreement of the raw beef consumers on the exposure to diseases from raw beef, the fatality of diseases originated from raw beef, the benefits of raw beef-eating, the effect of spices and alcohol on the raw beef borne pathogens, the effect of heating/cooling on raw beef borne pathogens, the contamination of raw beef with dangerous pathogens, the raw beef’s potential to transmit diseases to humans and the respondents’ belief in the safety of raw beef-eating were the items used for the assessment of raw beef consumers perception.

The data set prepared from the 34 questions and the dependent variables of choice, intention, and perception of raw beef consumers were analyzed using bivariate logistic regression with SPSS version 25.

### Data analysis

After the target sample size was collected, it was administered in Microsoft Excel 2013. Based on the answer of each choice, intention, and perception related questions, dependent binary variables were created for each choice, intention, and perception assessments of raw beef consumers. The participants whose answers were an indicator of raw beef-eating choice was categorized as “unfavourable choice” and whose answers were an indicator of not choosing raw beef-eating were grouped into favourable choice. Likewise, all the participants who intended to stop eating raw beef were grouped into favourable intentions, and those whose intentions was the opposite was categorized into the unfavourable intention category. In the same with choice and intention, the participants who perceive the health risks of eating raw beef were grouped into favourable perceptions, and those who perceive the opposite were categorized into unfavourable perceptions.

Based on the *p*-value of the logistic regression, the predictive explanatory variables for the result, favourable choice or unfavourable choice, favourable intention or unfavourable intention, and favourable perception or unfavourable perceptions were identified. The investigations of the participants’ choice, intention, and perception were conducted in three steps. The first step was assessing the relationship between potential predictor variables with the participants’ choice, intention, and perception one by one. Secondly, the relationship for the potential confounding effects was adjusted. Finally, the possibility of an interaction effect among the variables was considered.

To have initial insight into the structure of the data, cross-tabulations were used in SPSS version 25. From this basic descriptive tool, it is possible to see the proportions of each response category, which were indicative of the level of participants’ choice, intention, and perception of raw beef-eating.

After descriptive investigations using crosstabs, the association between the dependent binary variables (choice, intention, and perception) and each predictive variable was conducted. Probability values were used to see the association between these dependent binary variables and predictive variables (variables produced from each question). The effect levels of predictive variables on choice, intention, and perception of the participants were shown by the odds ratio (OR 95%CI).

## Results

### General information on raw beef consumers’ eating habits

For this study, a total of 570 raw beef consumers were interviewed. As indicated in Table 1, the majority (76%) of the participants have three meals per day and 43% of the raw beef consumers eat their meal at regular intervals of time (breakfast, lunch, and dinner). All the participants were raw beef consumers and a considerable number (31%) of them did not remember how they started eating raw beef. All (100%) of the participants were added spice on raw beef and 42% of them had a practice of drinking alcohol after raw beef-eating to facilitate metabolism, killing beef-borne pathogens, and for the sake of attaining optimum mood.

**Table 1:** General information of raw beef consumers on raw beef eating habits

Questions	Responses	Number (***n*** = 570)	Percent
How many meals do you usually consume daily?	1 meal	8	1
	2 meals	95	17
	3 meals	432	76
	4 meals	35	6
Do you consume meals at a regular time?	No	124	22
	Yes, some of them	199	35
	Yes, all of them	247	43
How did you start eating raw beef?	Peer pressure	129	23
	Habit from the ancestors	157	28
	I do not remember	174	31
	Intentionally started	110	19
Do you add spices to the raw beef before you consume it?	Yes, but only sometimes	338	59
	Yes, always	232	41
What type of spice do you add to raw beef before you consume it?	Pepper	81	14
	Chili peppers	219	38
	A mixture of spices	270	47
What did you do after you consume raw beef?	Drinking alcohol	240	42
	Taking tea and coffee	158	28
	Physical exercise	9	2
	Other	163	29
What is your reason for the post raw beef-eating actions you mentioned in the previous question?	Increasing metabolism	218	38
	Killing microbes	10	2
	To have a good feeling	137	24
	It is my habit	144	25
	No reason	61	11

### Consumers’ choice of raw beef eating

In statistical analysis, the predicted probabilities for the consumers’ choice of raw beef consumption were unfavourable. The predictor variables of raw beef consumers’ favorite meat-preparation type, feeling of raw beef consumers in the absence of raw beef eating, and the time of meal (breakfast, lunch, or dinner) for raw beef-eating were significantly associated (*P* < 0.05) with the pooled choice of raw beef consumers on raw beef-eating (Table 2).

**Table 2:** The bivariate logistic regression of predictor variables with the pooled consumers’ choice of raw beef eating

Questions	Responses	Percent (***n*** = 570)	P-value	OR (95% CI)
What is your preferred preparation type?	Heated	68	0.0001	15.021
	Raw meat	32		
What type of raw meat do you usually consume?	Beef	89	0.096	0.457
	Beef and Mutton	11		
What is your reason for eating raw beef?	Easy to prepare	10	0.660	1.046
	It was Cheap	2		
	Cheers me up	34		
	Keeps me healthy	16		
	My traditional food	25		
	High nutrient level	13		
What did you feel if you did not eat raw beef?	Nothing	76	0.0001	4.449
	Hunger	9		
	Uncomfortable	15		
How often do you eat raw beef?	1–3 times a month	19	0.991	0.999
	Once a day	25		
	Once a week	13		
	Few times a day	7		
	A few times a week	20		
	Only in the holidays	16		
In which of your meals do you prefer to eat raw beef?	Breakfast	12	0.0001	1.673
	Launch	68		
	Dinner	8		
	As part of all meals	12		

*OR* Odds ratio, *CI* Confidence interval

The odds of raw beef consumers’ meat-preparation preferences were 15 times greater than favourable choice than being unfavourable. Likewise, the odds of the raw beef consumers’ feeling in the absence of raw beef-eating was 4.5 (Table 2).

### The intention of raw beef consumers towards changing raw beef eating habit

The raw beef consumers’ intention to reducing raw beef-eating, intention in improving raw beef safety knowledge, and difficulty to change raw beef-eating habits were significantly associated (*P* < 0.05) with the pooled intention of changing a raw beef eating habit (Table 3).

**Table 3:** The bivariate logistic regression of predictor variables with the pooled intention of avoiding the raw beef eating habit

Questions	Responses	Percent (***n*** = 570)	P-value	OR (95% CI)
Are you currently intending to reduce/stop eating raw beef?	No	59	0.0001	0.098
	Yes, for a medical reason	8		
	Yes, by personal decision	34		
How would you describe your knowledge about the health risk of raw beef consumption?	Insufficient	24	0.071	0.749
	Sufficient	48		
	Good	24		
	Very good	4		
Are you intending to improve your knowledge on the health risk of raw beef consumption?	Highly interested	19	0.0001	2.644
	Moderately interested	33		
	In dilemma	18		
	Not interested	26		
	Strongly not interested	3		
Will you stop /reduce eating raw beef if you know its health impact?	Never	15	0.368	1.141
	May be	39		
	Immediately stop	29		
	Need time to decide	17		
How easy is it to change your raw beef eating habit?	Very easy	12	0.0001	0.387
	Easy	47		
	Unsure	21		
	Not easy	12		
	Impossible	7		
What prevents you from stopping eating raw beef?	Good for my health	47	0.306	0.841
	I am dependent on it	20		
	It has no health risk	17		
	I did not have another alternative	16		

*OR* Odds ratio, *CI* Confidence interval

The predicted probability in Table 3 is of membership for the unfavourable intention on raw beef consumption. The odds of unfavourable intention in reducing raw beef eating, and the difficulty of changing raw beef-eating habits were 0.098 and 0.387, respectively. On the contrary, the odds of raw beef consumers’ unfavourable intention towards both raw beef safety, and improving raw beef safety knowledge were 2.6 times of favourable intention (Table 3).

### The raw beef consumers’ perception of the safety of raw beef eating

Among the 8 questions forwarded to the raw beef consumers for assessing their perception of raw beef-eating, only 3 questions (the disease exposure from raw beef eating, the advantage and disadvantage of raw beef consumption, and the effect of cooking and cooling on the pathogens in raw beef) were significantly associated (*P* < 0.05) with the pooled perception of raw beef consumers. The unfavourable perception of raw beef consumers towards reducing the health risk from raw beef-eating was 2.7 times greater than the favourable perception (Table 4).

**Table 4:** The bivariate logistic regression of predictor variables with the pooled perception of raw beef consumption health risk

Questions	Modalities	Percent (***n*** = 570)	P-value	OR (95% CI)
Eating raw beef can expose the consumers to diseases.	Strongly disagree	6	0.0001	0.608
	Disagree	22		
	Neutral	5		
	Agree	58		
	Strongly agree	9		
The disease that originated from raw beef can be fatal to consumers.	Strongly disagree	20	0.076	1.198
	Disagree	54		
	Neutral	12		
	Agree	11		
	Strongly agree	3		
The benefits of consuming raw beef are greater than the health risks.	Strongly disagree	10	0.0001	2.745
	Disagree	36		
	Neutral	23		
	Agree	26		
	Strongly agree	5		
The spices added to the raw beef and the alcohol drunken after raw beef consumption can kill the pathogens.	Strongly disagree	11	0.080	0.850
	Disagree	18		
	Neutral	12		
	Agree	51		
	Strongly agree	7		
Cooking and/or cooling meat before consumption kills the beef-borne pathogens.	Strongly disagree	1	0.002	0.833
	Disagree	2		
	Neutral	5		
	Agree	61		
	Strongly agree	30		
The meat can be contaminated with dangerous pathogens along its value chain.	Strongly disagree	1	0.559	0.945
	Disagree	17		
	Neutral	49		
	Agree	26		
	Strongly agree	5		
The diseases from animals, persons, and the environment can transmit to humans through raw beef consumption.	Strongly disagree	7	0.916	0.991
	Disagree	27		
	Neutral	18		
	Agree	36		
	Strongly agree	12		
How do you believe about the safety of raw beef consumption?	Safe	32	0.129	1.206
	Unsafe	27		
	Neutral	25		
	I do not know	16		

*OR* Odds ratio, *CI* Confidence interval

### Summary of choice, intention, and perception of raw beef consumers

**Fig. 1:**
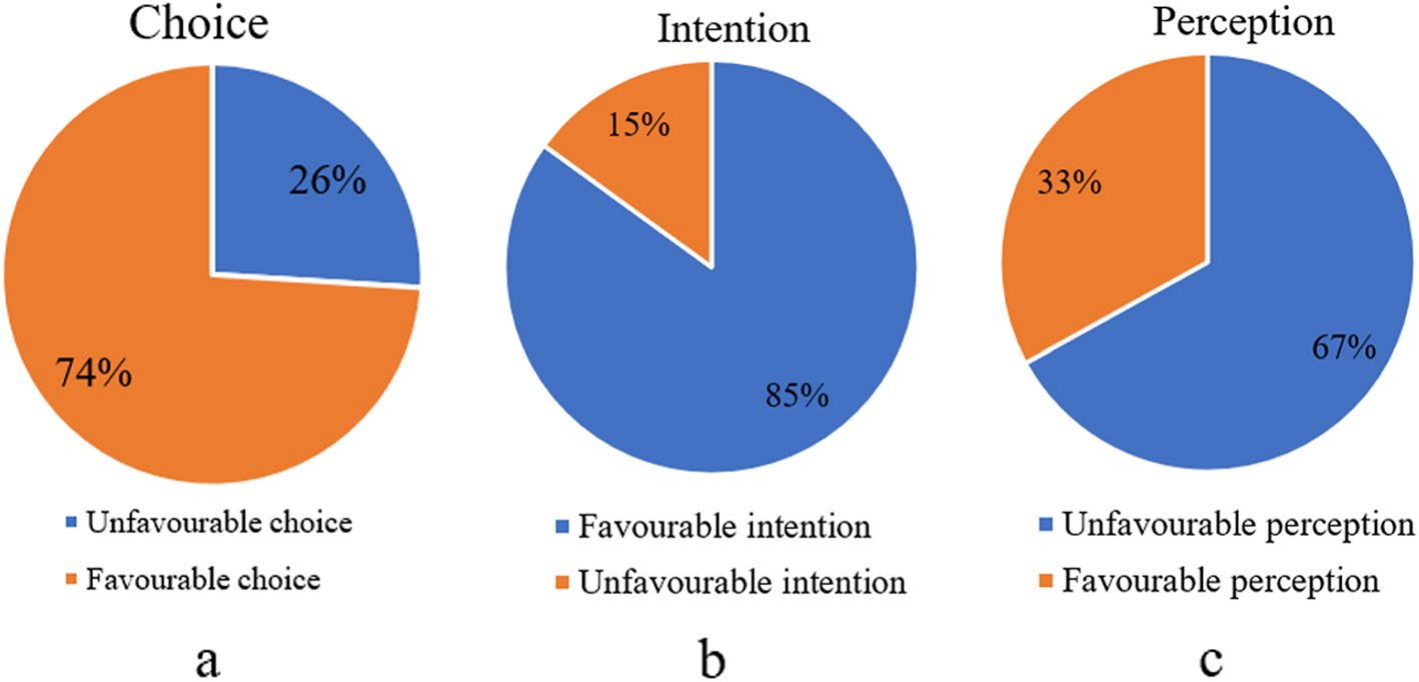
Summary of the raw beef consumers’ choice on raw beef-eating (**a**), intention to change raw beef-eating (**b**), and perception on the safety of raw beef-eating (**c**)

Based on the pooled variables of choice, intention, and perception, a considerable number (74%) of the raw beef consumers had favourable beef type choice (Fig. 1 a) and 85% of the raw beef consumers had favourable intentions to stop raw beef-eating habits (Fig. 1 b). In the contrary, majority (67%) of the participants had a unfavourable perception of the safety of raw beef consumption (Fig. 1 c).

### Demographic variables’ associations with consumers’ choice, intention, and perceptions

The predicted probabilities for choice, intention, and perception of the raw beef consumers in Table 5 are unfavourable choice, unfavourable intention, and favourable perception, respectively. Location, sex, marital status, health status, and educational status had a statistically significant association (*P* < 0.05) with the pooled choice of raw beef consumers on raw beef-eating. Sex, marital status, health status, and educational status of raw beef consumers have a significant association with the pooled intention of raw beef consumers to stop raw beef eating while only the age of the participants had a significant association with the pooled raw beef safety perception of the raw beef consumers (Table 5).

**Table 5:** Association of the demographic variables with the pooled raw beef consumers’ choice on raw beef-eating, intention to change raw beef-eating, and perception about raw beef eating

Questions	Responses	Percent	Choice		Intention		Perception	
			P-value	OR (95% CI)	P-value	OR (95% CI)	P-value	OR (95% CI)
**Location**	Dessie	35.09	0.0001	0.570	0.116	0.844	0.522	0.934
	Kombolcha	26.32						
	Kemissie	17.54						
	Bati	12.28						
	Wereilu	8.77						
**Age**	18–35	54.91	0.451	0.843	0.947	1.015	0.003	1.894
	36–50	35.96						
	> 50	9.12						
**Sex**	Male	72.11	0.0001	3.402	0.005	2.827	0.216	0.702
	Female	27.89						
**Marital status**	Single	53.16	0.0001	2.633	0.022	1.692	0.549	0.851
	Married	42.28						
	Widowed	4.56						
**Health status**	Poor	3.16	0.0001	0.520	0.0001	0.475	0.643	0.915
	Good	57.19						
	Very good	28.60						
	Excellent	11.05						
**Weight status**	Under	3.16	0.956	0.992	0.364	1.126	0.128	0.740
	Normal	84.56						
	Over	8.60						
	Obese	3.68						
**Educational status**	Primary	8.95	0.006	1.651	0.013	1.593	0.077	0.682
	Secondary	37.37						
	Higher	53.68						

*OR* Odds ratio, *CI* Confidence interval

## Discussion

### Raw beef eating habit

All (100%) raw beef consumers were adding spices to raw beef before consumption due to the consumers’ belief in preventing diseases. As a confirmation of the raw beef consumers’ belief, the study [16, 17] conducted on “food spices” revealed that spices are potent to treat different allergic, chronic, and infectious diseases. In addition to the spices’ effect on pathogens, the raw beef consumers have added spices for having deliciousness/good flavor. Likewise, the study on food spices showed that spices are added for making the food tasty and for other health benefits [16, 18]. Not only did adding spices to raw beef, but 42% of consumers were also drinking alcohol after they ate raw beef for killing pathogens and to bring a bright mood. Comparably, the study [19] done on the benefit of alcohol drinking after food showed that alcohol can kill pathogens that were ingested together with food. Even if different studies confirmed the anti-pathogens effect of spices and alcohol, their effect could be depending on the type of pathogen and the dose of the spice and alcohol. As a result, eating raw beef with the guarantee of spices in it and the alcohol drunken after raw beef consumption can cause a substantial health crisis [20, 21].

### Consumers’ choice of raw beef eating

Only 32% of the raw beef consumers had always prepared raw beef and the remaining 68% of consumers usually eat processed meat (cooked, roasted, fried, and stewed), and they occasionally eat raw beef. Comparable with the present finding, the study [22] conducted in eastern Asian countries showed that meat consumers had a variety of meat preparation preferences that ranges from eating raw meat to diverse types of processed meat. A substantial number (89%) of raw beef consumers prefer to eat raw beef than other types of raw meat (mutton, fish, or chicken). On the contrary, a larger number of consumers prefer to eat raw fish than other types of raw meats in Vietnam [23]. This raw meat type preferences might be due to differences in the type of available food (farming systems) in different geographic locations, and eating habit differences. These differences intern results in different food type dependencies of consumers.

In the absence of raw beef consumption, only 15 and 9% of the raw beef consumers had feelings of uncomfortable and hunger, respectively. This means that 24% of the raw beef consumers had favourable eating choice. Like the feeling of consumers with unfavourable beef eating choice, dependent consumers showed discomfort, hunger, sadness, and complicated mood in the absence of the intended food item [24].

A quarter (25%) of the raw beef consumers in this study had a practice of eating raw beef once per day and 16% of the participants had a practice of eating raw beef during holidays only. As described by the study on food addiction and an eating disorder, the frequent consumption of a specific type of food is a sign of food addiction [25].

The preferred meat preparation type, the feeling of the absence of raw beef consumption, and the raw beef-eating time (lunch, breakfast, or dinner) were significantly associated (*P* < 0.05). Raw beef consumers were 15 times preferred to eat heated meat (favourable eating choice) than eating raw beef (unfavourable choice). In the same way, raw beef consumers felt 4.4 times nothing (favourable) than other raw beef addiction feelings (hunger and uncomfortable).

Lunch is the most important and never omitted meal of the day in Ethiopian people. Consumers are interested in having the food items they prefer most in their important type of meal. In addition to this justification, Aoyama, and Shibata [26] confirmed that the consumers who eat food items composed of protein and lipid showed a postprandial dependency on lunchtime. As a result, the choice of raw beef eaters depends on eating raw beef at lunchtime or not at lunchtime (breakfast and/or dinner). Raw beef consumers were 1.6 times more eating raw beef at lunchtime (unfavourable beef eating choice) than not at lunchtime (favourable beef-eating habit). Comparable with the present finding, the time of the meal and the consumers’ mood in the absence of target food in their important meal were the signs of food specific food choice [27].

### Intention to change raw beef eating

About a quarter (27%) of the raw beef consumers believed that eating raw beef is not safe, and this finding agrees with [28–30]. A larger number (32%) of the participants believed that raw beef-eating is safe for their health. In contrast with the present finding, the study [29, 30] on raw beef safety indicated that eating raw beef exposes consumers to dangerous pathogens.

A larger number (52%) of the raw beef consumers were interested in improving their knowledge on the safety of raw beef eating while 29% of the consumers were not interested. Comparable to the present finding, many participants were interested in improving their understanding of food safety [31, 32]. This finding showed that if awareness creation on raw beef-eating is done the raw beef eating habit of raw beef consumers can be changed. Around half (47%) of the raw beef consumers continued consuming raw beef because they believe that raw beef’s health benefit is higher than processed meat (cooked, stewed, roasted, and fried). This finding is supported by the study conducted on the nutritional quality of meat [33, 34], which proves that meat processing reduces the nutritional and organoleptic quality of meat. Changing their raw beef eating habit is easy for 59% of the raw beef consumers and difficult for 19% of them. The interest of the raw beef consumers in changing their eating habits is a good standing point to sensitize them about the health risk [20, 21] of raw beef-eating and then to shift their imprudent trend to a healthy eating style (consumption of processed meat).

The raw beef consumers’ intentions regarding their beliefs on raw beef safety were 2.6 times unfavourable intention with unsafe, neutral and I do not know responses than favourable intention with a safe response. On the contrary, their interest in improving their food safety knowledge was 2.6 times more favourable intention with highly and moderately interested responses than unfavourable intention with not interested, strongly interested, and in dilemma responses. In the opposite to the present finding, the study on “consumers’ intention and knowledge of food safety” showed that consumers were very flexible to change their eating habits if they are properly inducted about the possible consequences of their practice [35]. These agreements might be due to educational, religious, and cultural differences in the study populations. The raw beef consumers were 0.38 times more favourable intentions (changing their eating habits easily and very easily) than unfavourable intentions with unsure, not easy, and impossible responses. This finding showed that the raw beef consumers had favourable beef eating choice and they were interested in changing their eating habits if special assistance like awareness creation is performed. Comparably, the study done on “mindfulness, mindful eating and intuitive eating in changing eating behaviors” [36] indicated that it is possible to change the consumers’ eating habits easily if they are not strongly addicted, and committed.

### Perception towards raw beef eating

More than half (58%) of the raw beef consumers agreed that raw beef-eating can expose them to foodborne diseases, and 54% of the raw beef consumers agreed that the diseases from raw beef can be fatal. Comparably, the study conducted on food-borne zoonoses [37–39] showed that raw beef is the most important source of pathogenic micro-organisms and its fatality rate is dependent on agent, host, and environmental factors [40, 41].

More than half of the raw beef consumers (51%), were perceived that the spice in the raw beef and the alcohol drunken after raw beef-eating can able to kill raw beef-borne pathogens. Similarly, the study done on the effect of spices and alcohols [16, 17] on food-borne pathogens indicated that adding spices in raw foods can kill microbes in them. 61% of the raw beef consumers perceived that heating/cooling of raw beef before consumption can reduce the beef-borne pathogens. Similarly, other research findings [42] recommended that meat processing (cooking, stewing, cooling, or roasting) kills/inhibits multiplication. As the study on the effect of heating on food-borne pathogens [43] described that some spores of microbes are resistant to heat treatment and cooking for a long time with a high-temperature level is recommended.

A larger number (48%) of participants perceived that the pathogens from cattle and the environment can be transmitted to raw beef consumers through raw beef-eating. This finding agrees with other findings [44–46] conducted on zoonotic and communicable diseases. The raw beef consumers’ perception of the raw beef-borne disease exposure, the cons and pros of raw beef consumption, and the effect of cooking/cooling of raw beef in the reduction of pathogens were significantly associated (*P* < 0.05) with the perception of raw beef consumers and each variable had the odds of 0.61, 2.75, and 0.83, respectively. The raw beef consumers’ favourable perception of the cons and pros of raw beef-eating was 2.75 times greater than their unfavourable perception. Comparable with the present finding the study conducted in Brazil [47] showed that consumers’ perception of food safety and nutritional quality of food items were significantly associated with their thoughts on the advantage and disadvantages of eating raw food items.

### The effect of demographic variables on choice, intention, and perception of raw beef consumers

All sex, marital status, health status, and educational status of the raw beef consumers were significantly associated (*P* < 0.05) with the consumers’ choice of raw beef consumption. The consumers with good health status had 0.5 times less unfavourable food choice than participants with other types of health statuses. Male raw beef consumers were 3.4 times unfavourable food choice than female and, raw beef consumers with single marital status had 2.6 times unfavourable food choice than married and widowed. On the other hand, raw beef consumers with higher educational status had 1.7 times less favourable food choice than raw beef consumers with primary and secondary school educational status. Contrary to the present finding, the age of the participants showed a significant association with the choice and intention of consumers [48]. Alike the present finding, the marital statuses of consumers were significantly associated (*p* > 0.05) with their beef type choice and intention on eating raw beef [47]. The location of the raw beef consumers was significantly associated with the choice of raw beef consumers on raw beef consumption. This can be elaborated as, the raw beef consumers in Dessie city were 0.6 times favourable food choice than other city participants.

Alike the choice of the raw beef consumers, sex, marital status, health status, and educational status of the raw beef consumers were significantly associated (*P* < 0.05) with the intention of consumers to stop raw beef-eating. Male consumers had 2.8 times more favourable intention to change their raw beef-eating habits than female and unmarried/single consumers had 1.69 times favourable intention to change their imprudent raw beef-eating habits than married and widowed consumers. Similarly, consumers with favourable health status had 0.47 times more favourable intention to change a raw beef eating habit than other categories and consumers with higher educational status had 1.59 times favourable intention to change their raw beef eating habit. Comparable with the present finding, the sex and educational status of consumers in Turkey [48] showed significant association with the intention of reducing imprudent eating habits.

Only the age of the participants had a significant association with the perception of raw beef consumers on the safety of raw beef-eating. The adult age groups (18–35 years) had 1.89 times unfavourable perception of the safety of raw beef-eating than other age groups (36–50 and > 50 years). Likewise, the study in Brazil showed that the age of the consumers was significantly associated with their perception of food safety [47]. Contrary to the present finding, educational status, marital status, and sex of the participants were significantly associated with the food safety perception of consumers. These differences might be a result of cultural, educational, and socio-economic differences.

## Limitations

The study might be liable to social desirability and recalls bias. In addition, the nature of the study design (cross-sectional) can influence the cause-and-effect relationship of the predictor variables and the dependent binary variables (choice, intention, and perception) of the raw beef consumers. Since there was no study done before the present assessment, it was not possible to compare numerical figures with other study findings.

## Conclusion

The raw beef eating habit of Ethiopians can create a favourable condition for the transmission of pathogens from contaminated meat to raw beef consumers. Even if many raw beef consumers had favourable beef eating choice, but some of them were addicted to it. The majority of the raw beef consumers intended to change their raw beef-eating trend if they know the health crises from it. The independence of raw beef eaters from eating raw beef and the consumers’ interest to stop/reduce raw beef-eating are the excellent opportunities to change the eating habits of raw beef consumers. From this description, it is possible to understand that raw beef consumers can shift from raw beef consumption to processed (cooked, stewed, roasted, and fried) beef with minimum effort. The perceptions of many raw beef consumers on the safety of raw beef consumption were unfavourable. Based on the current finding, it is recommended to conduct consecutive awareness creation to change the raw beef consumers’ eating habits. By increasing the raw beef consumers’ understanding of the health risk of raw beef-eating, it is possible to change the intention and perception of raw beef consumers towards reducing raw beef-eating and increasing consumers’ understanding of the health risks of raw beef consumption.

## Data Availability

The data used to support the findings of this study are included in the article in the frequency table. In addition to this, the whole data set that is used to analyze the habit, choice, intention, and perception of raw beef consumers is attached as supplementary materials.
